# Exploring the heterogeneous morphometric data in essential tremor with probabilistic modelling

**DOI:** 10.1016/j.nicl.2022.103283

**Published:** 2022-12-06

**Authors:** Thomas A.W. Bolton, Dimitri Van De Ville, Jean Régis, Tatiana Witjas, Nadine Girard, Marc Levivier, Constantin Tuleasca

**Affiliations:** a Department of Clinical Neurosciences, Neurosurgery Service and Gamma Knife Center, Centre Hospitalier Universitaire Vaudois, 1011 Lausanne, Switzerland; b Department of Radiology, Centre Hospitalier Universitaire Vaudois, 1011 Lausanne, Switzerland; c Institute of Bioengineering, Ecole Polytechnique Fédérale de Lausanne, 1202 Geneva, Switzerland; d Department of Radiology and Medical Informatics, University of Geneva, 1202 Geneva, Switzerland; e Stereotactic and Functional Neurosurgery Service and Gamma Knife Unit, Assistance Publique-Hôpitaux de Marseille, Centre Hospitalier Universitaire de la Timone, 13005 Marseille, France; f Neurology Department, Assistance Publique-Hôpitaux de Marseille, Centre Hospitalier Universitaire de la Timone, 13005 Marseille, France; g Department of Diagnostic and Interventional Neuroradiology, Centre de Résonance Magnétique Biologique et Médicale, Assistance Publique-Hôpitaux de Marseille, Centre Hospitalier Universitaire de la Timone, 13005 Marseille, France; h University of Lausanne (UNIL), Faculty of Biology and Medicine (FBM), 1015 Lausanne, Switzerland; i Signal Processing Laboratory (LTS 5), Ecole Polytechnique Fédérale de Lausanne, 1015 Lausanne, Switzerland

**Keywords:** Essential tremor, Multivariate Gaussian, Gaussian mixture model, Surface-based morphometry, Cortical thickness, Surface area, Mean curvature, Head tremor, Hand tremor, Heterogeneity

## Abstract

Essential tremor (ET) is a prevalent movement disorder characterized by marked clinical heterogeneity. Here, we explored the morphometric underpinnings of this cross-subject variability on a cohort of 34 patients with right-dominant drug-resistant ET and 29 matched healthy controls (HCs). For each brain region, group-wise morphometric data was modelled by a multivariate Gaussian to account for morphometric features’ (co)variance.

No group differences were found in terms of mean values, highlighting the limits of more basic group comparison approaches. Variance in surface area was higher in ET in the left lingual and caudal anterior cingulate cortices, while variance in mean curvature was lower in the right superior temporal cortex and pars triangularis, left supramarginal gyrus and bilateral paracentral gyrus. Heterogeneity further extended to the right putamen, for which a mixture of two Gaussians fitted the ET data better than a single one.

Partial Least Squares analysis revealed the rich clinical relevance of the ET population’s heterogeneity: first, increased head tremor and longer symptoms’ duration were accompanied by broadly lower cortical gyrification. Second, more severe upper limb tremor and impairments in daily life activities characterized the patients whose morphometric profiles were more atypical compared to the average ET population, irrespective of the exact nature of the alterations.

Our results provide candidate morphometric substrates for two different types of clinical variability in ET. They also demonstrate the importance of relying on analytical approaches that can efficiently handle multivariate data and enable to test more sophisticated hypotheses regarding its organization.

## Highlights

• Surface-based morphometry modelling in essential tremor (ET) patients and controls.
• Bimodal (not unimodal) distribution of morphometry data in the right putamen.
• Cortical thickness and gyrification variances differ in several regions in ET.
• Broadly lower cortical gyrification goes with more severe head tremor.
• More atypical patients compared to the average have more severe upper limb tremor.

## 1 Introduction

Essential tremor (ET) stands amongst the most prominent movement disorders, with recent estimates of 3.2 cases per 1000 individuals and up to 28.7 for subjects older than 80 years of age (Welton et al., 2021). It is primarily characterized by upper limb action tremor, present for at least 3 years, and sometimes completed by head, voice, or leg tremor (Haubenberger and Hallett, 2018). More subtle deficits are also increasingly recognized as ET symptoms, including oculomotor dysfunctions (Helmchen et al., 2003), sleep disturbances (Jiménez-Jiménez et al., 2021), executive function and memory impairments, as well as mood disorders and dementia (Bermejo-Pareja, 2011, Louis et al., 2019).

The brain deficits that underlie ET are an active area of research. Results from post-mortem investigations converge on the presence of various cerebellar abnormalities that are thought to progressively develop over the course of the disease (Louis and Faust, 2020). Recent simulation and animal experimentation studies have clarified that tremor maintenance depends on the timing and strength of synaptic communication within the cerebello-dentato-rubro-olivary network (Zhang and Santaniello, 2019, Pan et al., 2020) – see Ibrahim et al., 2021, Pan and Kuo, 2022 for reviews. Furthermore, tremor generation also involves the overlapping cortico-ponto-cerebello-thalamo-cortical loop (Haubenberger and Hallett, 2018), which has been frequently pinpointed by neuroimaging investigations – see Pietracupa et al. (2021) for a review.

A popular approach to investigate the structural underpinnings of ET is *voxel-based morphometry* (VBM), which quantifies local differences in grey matter concentration (Ashburner and Friston, 2000). VBM has revealed widespread cortical and cerebellar brain atrophy in ET (Quattrone et al., 2008, Benito-León et al., 2009, Bagepally et al., 2012, Tuleasca et al., 2017). Automated segmentation methods, which delineate the human brain’s different tissue types (Bermejo-Pareja, 2011, Louis et al., 2019), have enabled the additional quantification of subcortical and cerebellar volumes, evidencing the presence of broad alterations in ET (Cerasa et al., 2009, Pietracupa et al., 2019, Prasad et al., 2019).

At the cortical level, *surface-based morphometry* (SBM) enables the extraction of several morphometric features, including cortical thickness (CT), surface area (SA) and mean curvature (MC). SBM provides complementary information to VBM (Palaniyappan and Liddle, 2012, Goto et al., 2021). In ET, the standard deviation of voxel-wise CT within the right inferior parietal and fusiform areas was found to differ between ET subjects and healthy controls (HCs) (Serrano et al., 2017). Furthermore, more severe tremor correlated with lower CT in the right paracentral gyrus and left isthmus cingulate (Benito-León et al., 2018). Somehow surprisingly, however, although SA and MC have been the subject of extensive clinical investigations in other brain disorders – see, *e.g.*, Shaw et al., 2012, Wallace et al., 2013 or Madre et al. (2020), whether and how these features are altered in ET remains poorly known.

To conduct SBM analysis, it is customary to apply similar steps, in parallel, for each of the morphometric features under scrutiny. Thus, dependences between properties are not explicitly modeled, although CT, SA and MC are strongly inter-related, as they showcase complex interactions that reconfigure across the lifespan (Raznahan et al., 2011, Hogstrom et al., 2013, Wierenga et al., 2014, Schnack et al., 2015). In addition, we have recently shown that in ET, cross-feature dependences are altered in some regions (Bolton et al., 2022a). Taking such relationships into account through a multivariate analysis is thus essential to accurately characterize the disease.

Another critical factor that extends beyond SBM analysis alone pertains to the acknowledged heterogeneity in the disorder’s clinical presentation. It has been suggested for many years that ET might represent a family of diseases (Louis, 2005), a common clinical syndrome (Hopfner et al., 2016a, Hopfner et al., 2016b), to the point that the identification of ET subtypes is contemplated as a primordial research question for coming years (Welton et al., 2021). Standard group comparison approaches (for example, an ET vs HC *t*-test for a feature of interest) typically assume unimodal data distributions and quantify average group differences. Association to clinical symptoms in the ET group is then probed through a correlation coefficient, with the underlying assumption that a larger or lower value for the feature of interest is associated to a greater extent of symptoms (Passamonti et al., 2011, Fang et al., 2013, Fang et al., 2015, Gallea et al., 2015, Lenka et al., 2017, Benito-León et al., 2018, Muthuraman et al., 2018, Boscolo et al., 2020, Nicoletti et al., 2020). Stratifying patients into sub-groups as a function of symptoms, and subsequently conducting a group comparison (Chung et al., 2013, Wang et al., 2018, Li et al., 2020), operates under the same assumption.

**Fig. 1:**
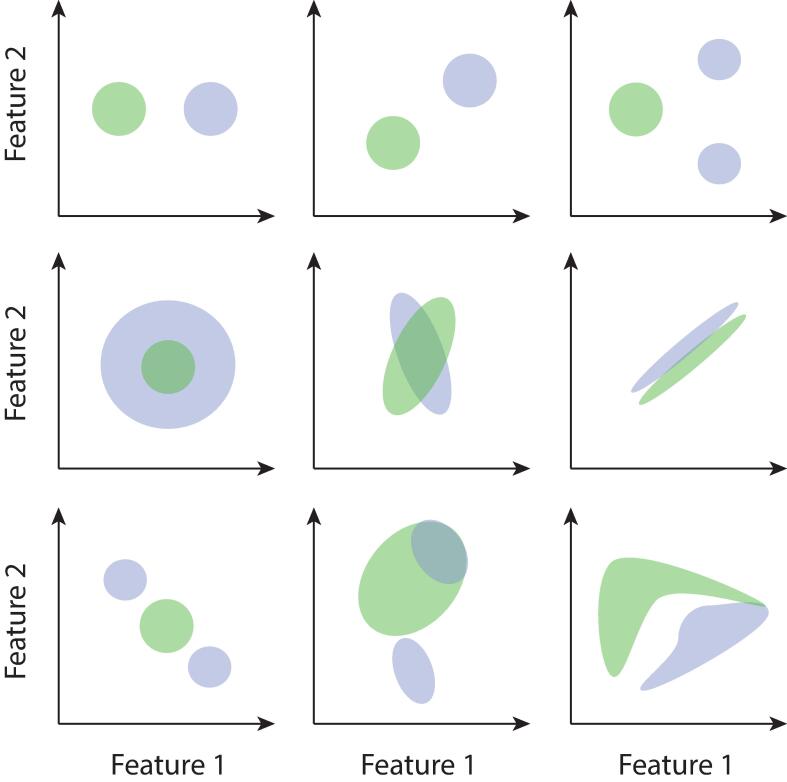
**Standard statistical approaches cannot accurately capture all forms of group differences.** Example cases in which two distributions of data points are schematically illustrated (green and blue) in a two-dimensional space. Standard univariate statistical group comparison approaches could only resolve a significant group difference for top row cases (and in the third one, would not capture the clustered nature of the data). The data may differ across groups in several more complex ways: in terms of variance (middle left), covariance (center), mean in a way that covariance prevents significance to be established with univariate tests (middle right). It may also exhibit a clustered nature such that group comparison captures no difference (bottom left), complex mixes of all the above (bottom middle), or nonlinear properties that Gaussian modelling cannot accurately capture (bottom right). (For interpretation of the references to colour in this figure legend, the reader is referred to the web version of this article.)

While these approaches have unequivocally advanced our understanding of ET, they remain blind to more complex types of data structure. Consider, for example, a scenario in which subsets of ET patients differ from healthy individuals in distinct ways: this may not be captured by a measurable average group difference. Fig. 1 schematically illustrates several potential complex data distribution scenarios.

In the present work, we assess to what extent complex data structures are present at the level of SBM features (CT, SA and MC) in a dataset of patients with ET and matched HCs. To do so, we resort to probabilistic modelling: to capture dependences between morphometric features, we model each group’s data with multivariate Gaussian distributions, which are parameterized not only by a mean vector (reflective of average morphometry values across subjects), but also by a covariance matrix (which jointly accounts for feature variance and cross-feature dependences). By comparing the fitting quality of a single Gaussian model to that of a mixture of Gaussians, we can explore whether a given brain region shows evidence for a multimodal data structure. By contrasting individual mean vector and covariance matrix coefficients across groups, we can determine whether ET patients may differ from HCs in terms of morphometric (co)variance on top of or instead of mean values. Finally, by quantifying the likelihood of the data to be a realization of a Gaussian process, we obtain a measure of conformity that weights different types of morphometric alterations equally.

## 2 Materials and methods

### 2.1 Subjects

This work included 34 right-handed patients (17 males) with drug-resistant essential tremor, who were 70.06 ± 9.12 (minimum: 49, maximum: 83) years old when initially assessed. They were neurologically evaluated by T.W., a neurologist specialized in movement disorders. They all had a clear diagnosis of ET based on consensus clinical criteria (Bhatia et al., 2018) and showed no other structural abnormalities upon 3 T magnetic resonance imaging (MRI). 23 had a familial history of ET, and symptoms’ duration was 35.53 ± 18.28 (minimum: 5, maximum: 61) years. All patients underwent Gamma Knife stereotactic radiosurgical thalamotomy of the ventro-intermediate nucleus of the thalamus, an intervention aimed at lowering tremor (Elaimy et al., 2010, Tuleasca et al., 2018a). Details on the procedure can be found in the Supplementary Material.

ET patients were compared to 29 age- and gender-matched HCs (69.93 ± 7.14 years old, 12 males). The Timone University Hospital Ethical Committee (ID-RCB: 2017-A01249–44) granted formal approval for this study (including by the Ethics Committee at national level, CNIL-MR-03). Individual consent was obtained from all subjects.

Several measures were used to clinically evaluate ET patients: Activities of Daily Living (ADL) from the survey designed by Bain and colleagues (Bain et al., 1993), Tremor Score on Treated Hand (TSTH) from the Fahn-Tolosa-Marín rating scale (Fahn et al., 1988), and head tremor (Tremor Research Group Essential Tremor Rating Assessment, from 0 to 3). Demographic and clinical data are summarized in Table 1.

**Table 1:** **Demographic and clinical details of the subjects.** For healthy controls (HCs) and ET patients, values are reported as mean ± standard deviation, with minimum, median and maximum into squared brackets. Significant statistical comparisons are highlighted in bold. M: male; F: female; ADL: activities of daily living; TSTH: tremor score on treated hand.

Variable	HC	ET	*p*-value
N	29	34	n.a.
Age [years]	69.93 ± 7.14 [59,69,83]	70.06 ± 9.12 [49,72,83]	*t_66_* = -0.06, *p* = 0.95
Gender [M:F]	12:17	17:17	n.a.
ADL	n.a.	29.59 ± 11.39 [13,28.5,49]	***t_66_* = 8.57, *p* = 2.48**∙**10^-12^**
Head tremor	n.a.	1 ± 0.85 [0,1,2]	***t_65_* = 2.16, *p* = 0.035**
TSTH	n.a.	20.41 ± 5.53 [8,20.5,30]	***t_66_* = 8.69, *p* = 1.52**∙**10^-12^**
Symptoms’ duration [years]	n.a.	35.53 ± 18.28 [5,33,61]	n.a.

### 2.2 Data acquisition

Native T1-weighted structural images were acquired for all subjects on the same head-only 3 T machine (SIEMENS SKYRA, Munich, Germany, 32-channel receive-only phase-array head coil). For ET patients, data was collected both at baseline and one year after thalamotomy. The acquisition parameters were as follows: TR/TE = 2300/2.98 ms, isotropic voxels of 1 mm^3^, 160 slices. Scanning was performed in a drug-naïve state (drugs having been stopped at least 3 days beforehand).

### 2.3 Data processing

#### 2.3.1 Extraction of morphometric features

Freesurfer (Fischl, 2012) was used to extract cortical thickness (CT), surface area (SA) and mean curvature (MC) from structural MR images for a set of *P*_cort_ = 68 cortical regions. Briefly, following linear registration to MNI space and bias field removal, the image at hand is skull-stripped (Ségonne et al., 2004), and voxels are classified as belonging to white matter or to another tissue category based on intensity and direct neighborhood. Following the separation of hemispheres and the removal of the cerebellum and subcortex, the interface between the white and gray matters and the pial surface are located. Local estimates of CT, SA and MC can then be extracted (Fischl and Dale, 2000; see Dale et al., 1999, Fischl et al., 1999 for more details). These voxel-wise measurements are eventually converted into P_cort_ regional values per morphometric measure, using the Desikan-Killiany atlas (Desikan et al., 2006). For each cortical brain region, a three-dimensional data point is thus obtained per subject, and our framework (see *Modelling framework* section below) models the distribution of these data points within each group (HC or ET).

In addition to the *P*_cort_ = 68 cortical brain regions, we also extracted regional volume for *P*_noncort_ = 19 non-cortical areas, including the cerebellum and subcortical nuclei, using *Freesurfer*’s automatic subcortical segmentation approach (Fischl et al., 2002). Our framework can also be seamlessly leveraged on this unidimensional data. Supplementary Table 1 summarizes all the brain regions considered in this work.

#### 2.3.2 Regression of covariates of no interest

To account for the confounding impacts of age, gender and total grey matter volume in our analyses, a mixed-effects model strategy was employed. We selected this approach because although the present work only focuses on the morphometric profiles of ET patients in their baseline state, MR imaging was also performed for each subject one year after Gamma Knife stereotactic radiosurgical thalamotomy of the ventro-intermediate nucleus of the thalamus. As mixed-effects models can account for within-subject variance on top of cross-subject variability in longitudinal data, we reasoned that it would be the most accurate way to describe our complete dataset.

For each region, each morphometric feature (CT, SA or MC for cortical areas and volume for non-cortical ones) was modelled as:

$$M_{i,s}=\beta_{0}+\beta_{1}A_{s}+\beta_{2}G_{s}+\beta_{3}V_{s}+\overset{2}{\underset{k=1}{\sum }}\beta_{3+k}I{\left[C_{k+1}\right]}_{s}A_{s}+\beta_{5+k}I{\left[C_{k+1}\right]}_{s}G_{s}+\beta_{7+k}I{\left[C_{k+1}\right]}_{s}V_{s}+b_{0,s}+{∊}_{i,s}.$$

In the above, *M_i_*_,_*_s_* is the morphometric feature’s value for region *i* and subject *s*. The model includes an intercept (*β*_0_ coefficient) and considers the individual impacts of age (*A_s_*, *β*_1_ coefficient), gender (*G_s_*, male=0, female=1, *β*_2_) and total grey matter volume (*V_s_*, *β*_3_).

Regarding the impact of group (*C*_1_ to C_3_ for HC, ET before and after thalamotomy, respectively), *I*[*C_k_*]*_s_* is a dummy variable encoding whether subject *s* belongs to group *C_k_*. Because of the presence of an intercept term in the model, only two dummy variables are needed to account for three groups as here. We model the interactions between group and the other 3 factors (*i.e.*, we enable distinct extents of confounding impacts across groups, as summarized by coefficients *β*_4_ to *β*_9_).

The term *b*_0,_*_s_* is the random effect for subject *s* (a subject-specific intercept), which follows a normal distribution with mean 0 and standard deviation $\sigma_{b}$. Finally, ${∊}_{i,s}$ is the error term, following a normal distribution with mean 0 and standard deviation σ.

The residuals of the model (including random effects, as well as group effects since they were not explicitly modelled) were used for all subsequent analyses.

### 2.4 Modelling framework

#### 2.4.1 Multivariate Gaussian model

Let a multivariate data point $x_{s}\in R^{d\times 1}$, with *d* > 1 the number of dimensions at hand. If $x_{s}$ is a realization of a normal distribution, then we have:

$$p\left(x_{s}|\mu ,\Sigma \right)≜N\left(x_{s}|\mu ,\Sigma \right)=\frac{1}{\sqrt{2\pi \left|\Sigma \right|}}e^{-\frac{1}{2}{\left(x_{s}-\mu \right)}^{T}\Sigma^{-1}\left(x_{s-}\mu \right)}.$$

In the above, μ is the mean vector, and Σ the symmetric positive semidefinite covariance matrix, which jointly describes the variance along each dimension (diagonal elements), and the covariance across dimensions (off-diagonal elements).

For a collection of *S* independent data points **X**, the log-likelihood (LL) is given by:

$$LL_{N}\left(X|\mu ,\Sigma \right)=-\frac{1}{2}\overset{S}{\underset{s=1}{\sum }}\left(\ln\left(\sqrt{2\pi \left|\Sigma \right|}\right)+{\left(x_{s}-\mu \right)}^{T}\Sigma^{-1}\left(x_{s}-\mu \right)\right).$$

The best mean vector and covariance matrix estimators, in the log-likelihood sense, are the data’s arithmetic mean and covariance matrix. Denoting the estimated mean vector $\hat{\mu }$ and covariance matrix Σ^ from a training dataset, the LL of a previously unseen data point ***z*** can also be evaluated as LLNz|μ^,Σ^.

#### 2.4.2 Group difference assessment

Let two separate sets of data, each modelled as a multivariate Gaussian process, for which the mean vector and covariance matrix have been estimated (that is, a total of 2*d* + *d*(*d*-1)/2 parameters per distribution). For each parameter, we take the difference between both sets as statistic of interest.

To assess significance, the difference is re-estimated after randomly shuffling the data points across groups 10′000 separate times (*i.e.*, non-parametric permutation-based significance testing). The actual value is compared to the resulting null distribution to compute a *p*-value (two-tailed assessment).

#### 2.4.3 Gaussian mixture model

A dataset exhibiting a multimodal data structure can be represented as a Gaussian mixture model (GMM). We define *K* as the number of mixed Gaussians. If ***x***_s_ is a realization from a GMM, we have:

$$p\left(x_{s}|{\left\{\mu_{k},\Sigma_{k}\right\}}_{k=1,\cdots ,K},\pi \right)=\overset{K}{\underset{k=1}{\sum }}\pi_{k}N\left(x_{s}|\mu_{k},\Sigma_{k}\right).$$

In the above, each Gaussian is parameterized by a mean vector and a covariance matrix, while π is a *K*-element vector that summarizes the respective weighting of the Gaussians and satisfies $\sum_{k=1}^{K}\pi_{k}=1$.

The GMM can be solved with the expectation–maximization (EM) algorithm (Moon, 1996), as detailed in the Supplementary Material. Following convergence, the LL of a previously unseen data point ***z*** can be evaluated as:

LLGz=∑s=1Slog∑k=1Kπ^kNxs|μ^k,Σ^k.

#### 2.4.4 Model comparison

To accurately compare Gaussian and GMM representations of a dataset, we resort to leave-one-out cross-validation (CV), where the two models are estimated on a set of training data points, and the LL is computed on the left-out sample. The sum of the LLs on left-out samples across CV folds is taken as a metric of model quality.

#### 2.4.5 Brain/behavior associations

Partial Least Squares (PLS) analysis is a multivariate approach that extracts covariance relationships across two sets of modalities. There have been many neuroimaging reports leveraging this tool to jointly study imaging and behavioral markers (Meskaldji et al., 2016, Zöller et al., 2017, DuPre and Spreng, 2017, Kebets et al., 2019, Bolton et al., 2020, Griffa et al., 2022). We outline the key steps of PLS analysis below; for a more comprehensive description, the reader is pointed to Krishnan et al. (2011).

Let a matrix of imaging variables **I**, of size *S* × *M* (with *M* the number of variables), and a matrix of behavioral/clinical scores **B** of size *S* × *B* (with *B* the number of scores). We assume that *M* ≥ *B*. Both matrices are normalized across subjects, and the cross-covariance across both sets is then computed:

$$R=I^{T}B={\mathrm{U\Sigma V}}^{T}.$$

The second equality relies on Singular Value Decomposition (SVD) of the covariance matrix. **U** has size *M* × *B*, **Σ** is a diagonal matrix of size *B* × *B*, and **V** has size *B* × *B*. The first columns of **U** and **V** contain the *salience weights* associated to the first mode of covariance found in the data (*i.e.*, largest fraction of explained covariance). A large value for a given element indicates that the imaging variable/clinical score at hand strongly contributes to the cross-modality covariance. The second columns of **U** and **V** equivalently contain the salience weights associated to the second mode of covariance found in the data, and so on.

To assess whether the extracted modes of covariance are significant, each of the actual singular values is compared to a null distribution, generated by performing PLS analysis 10′000 times after shuffling subject labels for one of the two sets of variables. The fraction of cases in which null singular values exceed the actual one is taken as *p*-value. Since PLS analysis is a multivariate approach, no further statistical correction needs to be applied.

It is also important to determine whether the obtained salience weights are robust. For this purpose, bootstrapping is conducted, where 80 % of the original data is sampled 10′000 times with replacement. For each salience weight, a 99 % confidence interval (CI) is constructed, and weights for which zero does not fall within the CI are deemed significant.

## 3 Application to morphometric data

For cortical brain regions, we considered three-dimensional data points for each subject (*d* = 3), encompassing the CT, SA and MC values as first, second and third dimension. For non-cortical brain regions, we considered one-dimensional data points (regional volume, *d* = 1), where univariate Gaussians are fitted and only two parameters characterize a distribution (the mean and the standard deviation).

The HC and ET groups were modelled separately, and group comparison was performed between both distributions to assess ET-induced morphometric alterations. All reported *p*-values were Bonferroni-corrected for the number of analyzed regions (*P* = 87).

When comparing the multivariate Gaussian and GMM representations, given the limited number of available data points (*S* = 29 and 34), we restricted ourselves to bimodal modelling (*K* = 2), for which the number of parameters to estimate remains affordable. Leave-one-out CV was performed.

After determining the parameters of the HC and ET multivariate Gaussian distributions (${\hat{\mu }}_{\mathrm{HC}}$, Σ^HC, ${\hat{\mu }}_{\mathrm{ET}}$ and Σ^ET), to quantify how individual ET morphometric profiles stand out from those of the overall population of patients, we computed LLNzsET|μ^ET,Σ^ET, where $z_{s}^{\left(ET\right)}$ is the regional data for patient *s*. Higher and lower values denote more typical subjects and more “outlier” subjects, respectively.

We performed PLS analysis in two settings to evaluate the associations between the morphometric data and clinical symptoms. We considered clinical information (baseline ADL, TSTH and head tremor scores, symptoms’ duration and family history of ET) as behavioral variables. As imaging variables, we considered either the concatenated morphometric values across regions (for a total of 3*P*_cort_ + *P*_noncort_ variables), or the LL of ET samples to be realizations of the ET distribution (*P* variables).

## 4 Data use and implementation details

The data analyzed therein was already examined in two previous structural covariance analysis studies (Bolton et al., 2022a, Bolton et al., 2022b). These past works primarily focused on differentiating HCs from ET patients in terms of cross-regional statistical dependences at the group level, did not examine mean group differences in morphometry, did not disentangle the impacts of within-group variance and covariance in the analyses (indeed, Pearson’s correlation coefficient was then used instead of the present modelling strategy), and did not include direct associations with clinical scores. The present results instead provide insight into cross-subject variability, and how this morphometric heterogeneity relates to the ET symptomatology.

Colormaps for plotting were generated with the *cbrewer* toolbox https://www.mathworks.com/matlabcentral/fileexchange/34087-cbrewer-, and PLS analysis was performed with the *myPLS* toolbox (https://github.com/danizoeller/myPLS). All other analytical steps described above were performed with custom scripts and MATLAB2020b (MathWorks, Natick, USA).

All the scripts used in this work are freely available at the following link: https://github.com/TiBiUan/SCA_IndividualDifferences.git. The data that support the findings of this study are available from the corresponding author upon reasonable request.

## 5 Results

**Fig. 2:**
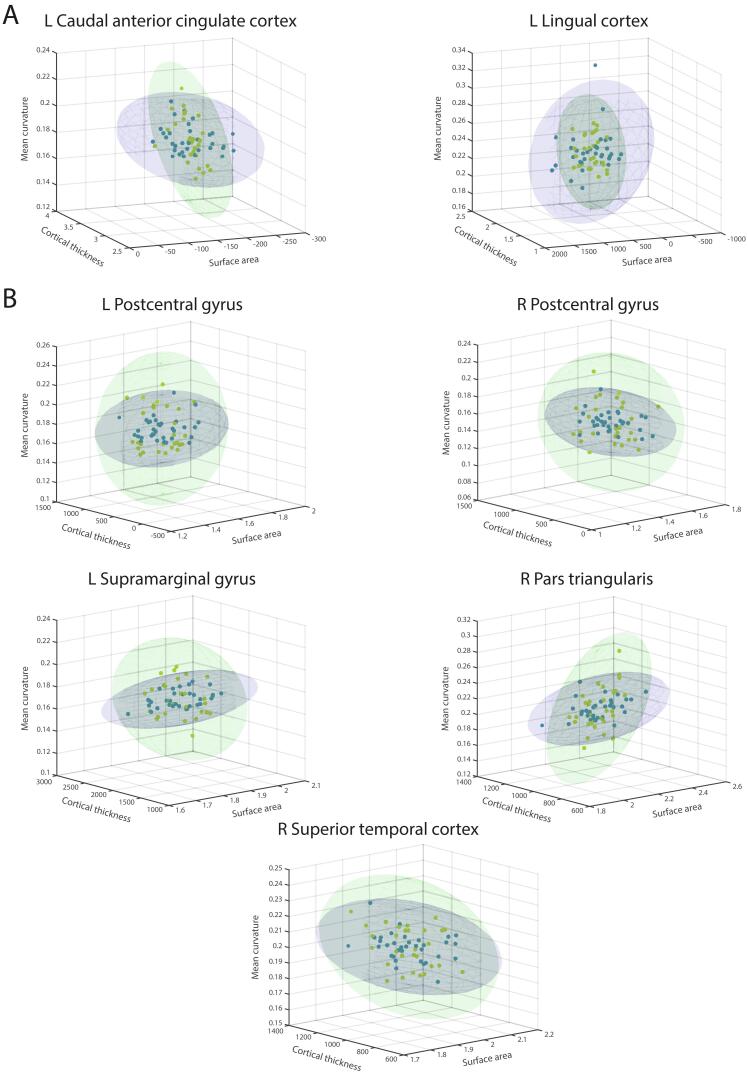
**Surface area and mean curvature variances differ between groups in specific brain regions.** For the cortical brain regions showing a significant HC vs ET group difference in surface area (A) and mean curvature (B) variance, representation of the HC (green) and ET (blue) data points in the three-dimensional (CT, SA, MC) space, and contours of the associated multivariate Gaussian fits with 68.2% of enclosed probability volume. L: left, R: right. (For interpretation of the references to colour in this figure legend, the reader is referred to the web version of this article.)

A standard analysis through *t*-tests with non-parametric permutation-based assessment of significance showed no significant HC vs ET group differences upon Bonferroni correction. To study the morphometric data in multivariate manner while assessing potential group differences in terms of (co)variance on top of mean values, multivariate Gaussian representations were contrasted between groups (Fig. 2). In line with the above analysis, following Bonferroni correction, there were no differences in terms of mean values. The same held true for covariance relationships. However, SA variance was significantly higher in the ET group in the left caudal anterior cingulate (Δσ^2^_SA_ = -885.362, *p* = 0.0035) and lingual (Δσ^2^_SA_ = -73990, *p* = 0.0313) cortices (Fig. 2A). At the same time, MC variance was significantly lower in the bilateral postcentral (Δσ^2^_MC_ = 3.5086, *p* = 0 and Δσ^2^_MC_ = 3.8631∙10^-4^, *p* = 0.007 [left and right, respectively]) and left supramarginal (Δσ^2^_MC_ = 2.0845∙10^-4^, *p* = 0) gyri, in the right pars triangularis (Δσ^2^_MC_ = 5.1511∙10^-4^, *p* = 0.007) and in the right superior temporal cortex (Δσ^2^_MC_ = 1.0548∙10^-4^, *p* = 0.0418; Fig. 2B).

**Fig. 3:**
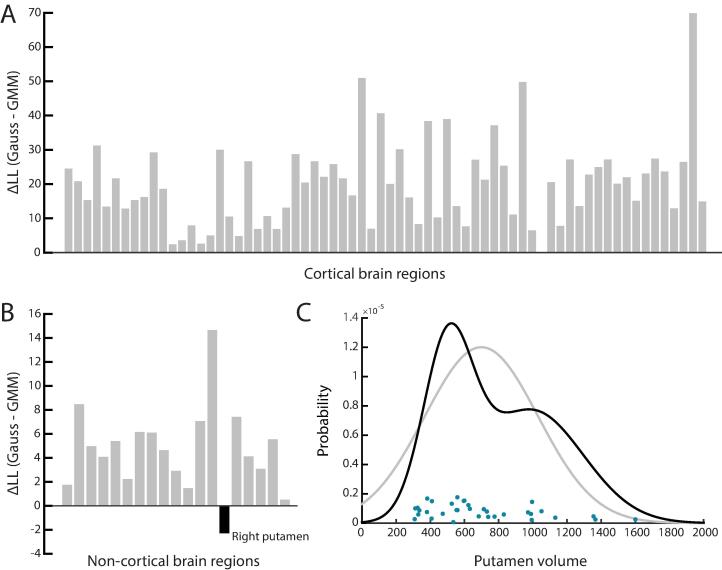
**A bimodal data organization is only seen in the right putamen.** (A) Difference in log-likelihood, across cortical brain regions, between cross-validated LLs for a multivariate Gaussian and a GMM. (B) Similar representation for non-cortical brain regions (for which univariate modelling of volume is performed). (C) Data distribution for the right putamen in the ET population (blue circles), where the GMM (black) offers a more accurate representation than a multivariate Gaussian (gray). (For interpretation of the references to colour in this figure legend, the reader is referred to the web version of this article.)

The differences in morphometric data variance seen across groups hint at varying extents of heterogeneity. To explore whether this would be due to a multimodal structure, in ET patients, the multivariate Gaussian representation was compared to a GMM. Across all cortical areas, the cross-validated LL was larger for the multivariate Gaussian model, providing no evidence for the presence of a clustered data structure (Fig. 3A). For non-cortical areas, the multivariate Gaussian representation was also more accurate in most cases, but the GMM representation fitted the data better in the right putamen for ET subjects ($LL_{G}$ = -245.33 > $LL_{N}$=-247.61; Fig. 3B/C).

**Fig. 4:**
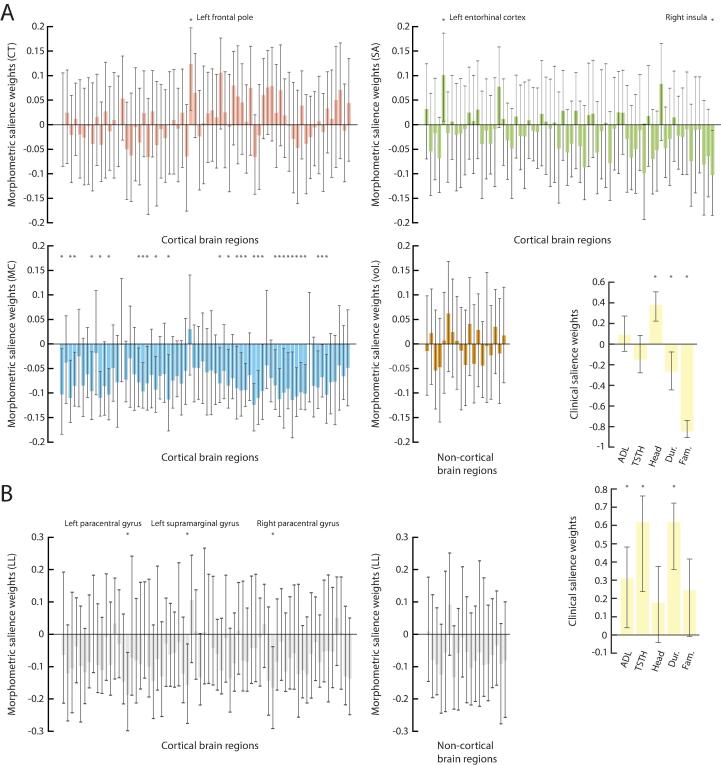
**Two types of association with clinical symptoms.** (A) Salience weights for clinical scores and imaging variables when three-dimensional morphometric data (CT, SA, MC) is considered. (B) Similar information when the regional LLs to result from the multivariate Gaussian ET distribution are used as imaging variables instead. Significance of salience weights (99% confidence interval not including zero) is highlighted by stars, and associated regions are labeled.

To explore the clinical relevance of the morphometric heterogeneity seen across ET patients, we used PLS analysis to extract relationships with clinical symptoms. When considering raw morphometric data, there was one significant mode of covariance (mode 1, *p* = 0.0282, 37.02 % of covariance explained; Fig. 4A). Subjects with more head tremor (99 % CI: [0.2224,0.5062]), no ET family history ([-0.9076,-0.74]) and lower symptoms’ duration ([-0.4438,-0.0759]) exhibited larger CT in the left frontal pole ([0.0291,0.1975]), higher SA in the left entorhinal cortex ([0.0071,0.1867]), lower SA in the right insula ([-0.1849,-0.0121]), and lower MC in a broad set of 30 regions detailed in Table 2.

**Table 2:** **Regions showing significant MC salience weights when linked to clinical scores.** CI: confidence interval.

Region name	99 % CI lower bound	99 % CI upper bound
Left Banks superior temporal sulcus	−0.1843	−0.0094
Left caudal middle frontal cortex	−0.16	−0.0332
Left cuneus	−0.1265	−0.0167
Left inferior temporal cortex	−0.1543	−0.0159
Left lateral occipital cortex	−0.1472	−0.0562
Left lingual cortex	−0.1549	−0.0501
Left pars triangularis	−0.1281	−0.0082
Left pericalcarine cortex	−0.1308	−0.0379
Left postcentral gyrus	−0.135	−0.0049
Left precentral gyrus	−0.1396	−0.0253
Left rostral middle frontal cortex	−0.1775	−0.0219
Right cuneus	−0.1194	−0.0078
Right fusiform cortex	−0.1307	−0.0115
Right inferior temporal cortex	−0.153	−0.0047
Right isthmus cingulate	−0.1469	−0.0178
Right lateral occipital cortex	−0.1277	−0.0394
Right lingual cortex	−0.1774	−0.0805
Right medial orbitofrontal cortex	−0.164	−0.0544
Right middle temporal cortex	−0.1539	−0.0006
Right pars opercularis	−0.1333	−0.0118
Right pars orbitalis	−0.1479	−0.0502
Right pars triangularis	−0.1716	−0.0093
Right pericalcarine cortex	−0.1322	−0.0175
Right postcentral gyrus	−0.1913	−0.0165
Right precentral gyrus	−0.148	−0.0484
Right posterior cingulate cortex	−0.1329	−0.0376
Right precuneus	−0.1345	−0.0421
Right superior frontal cortex	−0.1315	−0.0139
Right superior parietal cortex	−0.1082	−0.0009
Right superior temporal cortex	−0.1617	−0.0217

When instead considering the subject-wise LL to belong to the ET distribution as imaging variables, there was still one significant mode of covariance (mode 1, *p* = 0.0423, 65.1 % of explained covariance; Fig. 4B), but the highlighted relationship largely differed. This time, subjects with greater baseline ADL and TSTH scores ([0.0393,0.4816] and [0.2374,0.7609]), and larger symptoms’ duration ([0.3593,0.7225]) also showed broadly lower LL values across the brain, significantly so in the bilateral paracentral ([-0.2985,-0.0563] and [-0.2914,-0.0381], respectively left and right) and left supramarginal ([-0.2758,-0.0303]) gyri.

## 6 Discussion

### 6.1 Cortical gyrification associates with ET onset and head tremor

Here, on top of studying ET patients in terms of CT as previously done (Serrano et al., 2017, Benito-León et al., 2018), we considered SA and MC as complementary morphometric properties. In recent structural covariance studies (Bolton et al., 2022a, Bolton et al., 2022b), we showed that ET patients also differ from HCs in terms of these. The present results demonstrate that cortical gyrification also varies as a function of subject-specific ET symptomatology, as we found that individuals with more severe head tremor, a shorter duration of symptoms, and no familial history of ET also displayed lower cortical gyrification in somatomotor, visual, temporal, and frontal areas. Interestingly, this relationship was specific to tremor of the head, as the TSTH score (which instead quantifies the severity of upper limb tremor) was not significant in the association.

That the absence of an ET family history and a lower duration of symptoms are related is not surprising, since early-onset ET is more frequently reported in patients with a familial history (Louis, 2006, Hopfner et al., 2016a, Hopfner et al., 2016b). It has been supposed that late-onset ET may in fact reflect a dedicated “aging-related” tremor subtype (Deuschl et al., 2015), and our results position lower cortical gyrification as a morphometric correlate. In particular, the lower gyrification of frontal areas may relate to the increased risk of dementia documented in late-onset ET (Benito-León et al., 2006). The lack of cerebellar involvement also squares well with past histopathological work, in which cerebellar features did not differ between early-onset and late-onset ET (Kuo et al., 2016).

Patients with a shorter duration of symptoms and non-familial ET also exhibited more severe head tremor, consistently with previous studies (Lenka et al., 2015, Louis, 2016). The larger frequency of memory problems, affective disorders, and the overall more severe non-motor symptoms seen in patients with head tremor (Peng et al., 2020) may also be partly reflected in the weakened frontal, temporal and occipital gyrification that we observed. Interestingly, using functional MRI to contrast the amplitude of low frequency fluctuations (ALFF) between ET patients with and without head tremor, Wang and colleagues pinpointed the left middle frontal gyrus, right postcentral gyrus and right superior parietal lobule (Wang et al., 2018), all of which were captured in our analyses as well. The authors found ALFF in these areas to be lower in patients with head tremor, which is consistent with lower cortical gyrification as it likely reflects less efficient intracortical organization. The presence of additional significant regions in our analyses may result from the nature of the investigated modality (morphometry vs functional activation), and/or from the fact that we considered a population of drug-resistant ET patients who were on average 20 years older than those studied by Wang *et al*. (*i.e.*, more extensive disease progression).

In sum, we unraveled a morphometric pattern that contrasts non-familial ET patients with head tremor and short symptoms’ duration from familial cases without head tremor and with a longer duration of symptoms. Because PLS analysis extracts directions along which the clinical and morphometric data covary, our results favor a trait-like description of ET with head tremor (Louis, 2016) – *i.e.*, the phenotype may occur at varying intensities across individuals. We note that lowered gyrification extends to other movement disorders, as it was also observed in Parkinson’s disease (Zhang et al., 2013, Sterling et al., 2016).

### 6.2 Morphometric heterogeneity is linked to upper limb tremor

Our multivariate modelling approach showed that relying on average group differences alone is oversimplistic to accurately characterize ET-induced morphometric alterations. In fact, in our cohort of ET patients, there were no mean differences in morphometric features. However, within-group variance significantly differed on some occasions: for SA, it was higher in ET patients in the left caudal anterior cingulate and lingual cortices, while for MC, it was lower in the bilateral postcentral and left supramarginal gyruses, the right pars triangularis and the right superior temporal cortex.

The caudal anterior cingulate cortex and pars triangularis are implicated in cognition (Gray and Braver, 2002, Elmer, 2016), and associated group differences may thus relate to the non-motor deficits seen in ET patients (Bermejo-Pareja, 2011, Louis et al., 2019). The right superior temporal cortex contributes to spatial awareness (Karnath, 2001), the supramarginal gyrus to visual recognition (Stoeckel et al., 2009) and the lingual gyrus to low-level visual functions (Schankin et al., 2014). Their differential properties in ET strengthen the case for an involvement of the visual system in the disease (Archer et al., 2018, Tuleasca et al., 2018b, DeSimone et al., 2019, Tuleasca et al., 2019, Bolton et al., 2022b). Finally, the postcentral gyrus includes the somatosensory cortex, whose modulation in ET has been reported by previous functional studies (Fang et al., 2015, Lenka et al., 2017).

These group differences in morphometric variance hinted at the presence of ET-specific heterogeneity. Through PLS analysis, we discovered that this heterogeneity is in fact clinically relevant: indeed, more severe upper limb tremor, accompanied by greater impairments in activities of daily living and a larger duration of symptoms (likely because it also correlates with more severe tremor owing to the disease’s progression over time), were associated to a lower morphometric conformity to the distribution of ET subjects (*i.e.*, being an “outlier” with respect to the average). This was significant in the left supramarginal gyrus, also pinpointed by our group difference analysis, and in the bilateral paracentral gyrus, whose CT was, fittingly, negatively correlated to the severity of tremor in a previous morphometric study (Benito-León et al., 2018). Of note, albeit non-significantly, almost all other cortical regions also showed a similar effect direction (*i.e.*, lower conformity with more severe symptoms).

It is particularly interesting to notice that a link between upper limb tremor and morphometric features could only be drawn when an LL-based assessment was performed, as opposed to the use of raw morphometry data (which instead revealed morphometric correlates of head tremor, as discussed above). When computing the log-likelihood to be issued from a multivariate distribution, distinct types of morphometric alterations in the three-dimensional CT/SA/MC space can yield the same value if the “distance to the distribution” is equivalent (*i.e.*, if their log-likelihood is equal). Thus, we conclude that the morphometric correlates of upper limb tremor differ across ET patients in terms of their nature (higher vs lower than the average), and the respective contribution of their morphometric dimensions (CT, SA, and MC). In this, our results support the existence of ET subtypes in terms of the morphometric underpinnings of upper limb tremor.

Our findings emphasize the importance of leveraging analytical tools that can explicitly account for complex forms of cross-subject heterogeneity. In the present case, it was achieved by mapping distinct subtypes of morphometric alterations to an identical distance to the reference distribution. Our efforts complement those from other recent neuroimaging studies: in autism spectrum disorder, Hahamy and colleagues showed how heterogeneity in the spatial territories of specific functional areas across subjects could bias the results from group difference analyses (Hahamy et al., 2015). In the context of naturalistic paradigms, Finn *et al*. also proposed alternative measures to quantify cross-subject similarity for a behavioral score of interest, for which the distances are also weighted by the absolute behavioral score value – *e.g.*, two high-scorers may be deemed more similar than two low-scorers for an identical absolute difference in score (Finn et al., 2020).

Our comparison between multivariate Gaussian and GMM representations additionally enabled to assess whether cross-subject heterogeneity may manifest itself through separate clusters of data points. In most cases, this was not the case, as the multivariate Gaussian model outperformed the GMM. One could then assume that heterogeneity involves a continuum of alterations rather than a multimodal data distribution, but it should be emphasized that our comparison only considered *K* = 2 clusters, owing to the limited size of our dataset (*S* = 34 ET samples). Indeed, each additional cluster would require 7 additional parameters to be estimated (assuming *d* = 3). Future studies should clarify, on extended datasets, whether larger numbers of clusters may provide an optimal fit for some brain regions.

Interestingly, there was nonetheless one subcortical region for which the GMM did provide a better fit: the right putamen. Previous work revealed lower putamen ALFF in ET patients (Wang et al., 2018, Li et al., 2020), and larger local and global putamen functional connectivity with more severe tremor (Mueller et al., 2017). Additionally, several studies have reported that a subset of ET patients exhibit subtle deficiencies in dopaminergic receptors in the putamen (Shahed and Jankovic, 2007, Thenganatt and Jankovic, 2016), which is further evidence for multimodality of the area in ET.

Overall, our results demonstrate the presence of clinically relevant heterogeneity in a pool of drug-resistant patients with ET. The extent of upper limb tremor, daily living impairments, and the duration of symptoms are greater in patients that feature more distinctive morphometric patterns. The alterations do not follow a single direction in the multidimensional morphometric space, and to the exception of the right putamen, we found no evidence supporting a multimodal data distribution.

### 6.3 Limitations and future perspectives

As our analyses involved a relatively limited sample of 29 HCs and 34 patients with ET, our results should be viewed as preliminary until they can be replicated in future studies focusing on larger cohorts. It will also be important to consider a clinically broader set of patients, instead of only severely impacted drug-resistant individuals as here. Doing so is likely to reveal further types of heterogeneity in the data.

Our modelling approach could also be refined in several ways if larger datasets are considered: first, as alluded to above, the existence of multimodal data organization could then be probed for cluster numbers larger than 2. Second, more morphometric features could be jointly analyzed, as a larger sample size enables the accurate estimation of a larger array of parameters. Third, one could also consider to directly include cross-regional interactions within the modelling framework, instead of conducting independent parallel assessments that presently ignore the documented morphometric dependences between areas (Mechelli et al., 2005).

Finally, we believe that the application of our approach is warranted not only in ET, but also in other brain disorders for which heterogeneity has been an influential concept. This is for example the case of autism spectrum disorder, a neurodevelopmental condition that, like ET, exhibits a complex genetic basis (Persico and Napolioni, 2013) leading to clinical heterogeneity.

### CRediT authorship contribution statement

**Thomas A.W. Bolton:** Software, Validation, Formal analysis, Writing – original draft, Writing – review & editing, Visualization. **Dimitri Van De Ville:** Conceptualization, Writing – review & editing, Supervision. **Jean Régis:** Conceptualization, Investigation, Resources, Writing – review & editing, Supervision, Project administration. **Tatiana Witjas:** Investigation, Data curation, Writing – review & editing. **Nadine Girard:** Investigation, Data curation, Writing – review & editing. **Marc Levivier:** Conceptualization, Writing – review & editing, Supervision, Project administration. **Constantin Tuleasca:** Conceptualization, Resources, Writing – review & editing, Visualization, Supervision, Project administration, Funding acquisition.

## Declaration of Competing Interest

The authors declare that they have no known competing financial interests or personal relationships that could have appeared to influence the work reported in this paper.

## Data Availability

Data will be made available on request.
