# Supplementary material for: Exploring the heterogeneous morphometric data in essential tremor with probabilistic modelling
Source: Neuroimage Clin. 2022 Dec 6;37:103283. doi: 10.1016/j.nicl.2022.103283 (PMC9755240; doi:10.1016/j.nicl.2022.103283)
Supplement: Supplementary data 1 [file mmc1.pdf]

Supplementary Table 1: Regions of the considered atlas. All the regions analyzed in this work are listed alongside their index, and the hemisphere to which they belong.

**Index**	**Hemisphere**	**Name**
1	Left	Banks superior temporal sulcus
2	Left	Caudal anterior cingulate cortex
3	Left	Caudal middle frontal cortex
4	Left	Cuneus
5	Left	Entorhinal cortex
6	Left	Fusiform gyrus
7	Left	Inferior parietal cortex
8	Left	Inferior temporal cortex
9	Left	Isthmus cingulate
10	Left	Lateral occipital cortex
11	Left	Lateral orbitofrontal cortex
12	Left	Lingual cortex
13	Left	Medial orbitofrontal cortex
14	Left	Middle temporal cortex
15	Left	Parahippocampal gyrus
16	Left	Paracentral gyrus
17	Left	Pars opercularis
18	Left	Pars orbitalis
19	Left	Pars triangularis
20	Left	Pericalcarine gyrus
21	Left	Postcentral gyrus
22	Left	Posterior cingulate cortex
23	Left	Precentral gyrus
24	Left	Precuneus
25	Left	Rostral anterior cingulate cortex
26	Left	Rostral middle frontal cortex
27	Left	Superior frontal cortex
28	Left	Superior parietal cortex
29	Left	Superior temporal cortex
30	Left	Supramarginal gyrus
31	Left	Frontal pole
32	Left	Temporal pole
33	Left	Transverse temporal cortex

34	Left	Insula
35	Right	Banks superior temporal sulcus
36	Right	Caudal anterior cingulate cortex
37	Right	Caudal middle frontal cortex
38	Right	Cuneus
39	Right	Entorhinal cortex
40	Right	Fusiform gyrus
41	Right	Inferior parietal cortex
42	Right	Inferior temporal cortex
43	Right	Isthmus cingulate
44	Right	Lateral occipital cortex
45	Right	Lateral orbitofrontal cortex
46	Right	Lingual cortex
47	Right	Medial orbitofrontal cortex
48	Right	Middle temporal cortex
49	Right	Parahippocampal gyrus
50	Right	Paracentral gyrus
51	Right	Pars opercularis
52	Right	Pars orbitalis
53	Right	Pars triangularis
54	Right	Pericalcarine gyrus
55	Right	Postcentral gyrus
56	Right	Posterior cingulate cortex
57	Right	Precentral gyrus
58	Right	Precuneus
59	Right	Rostral anterior cingulate cortex
60	Right	Rostral middle frontal cortex
61	Right	Superior frontal cortex
62	Right	Superior parietal cortex
63	Right	Superior temporal cortex
64	Right	Supramarginal gyrus
65	Right	Frontal pole
66	Right	Temporal pole
67	Right	Transverse temporal cortex
68	Right	Insula
69	Left	Cerebellar white matter
70	Left	Cerebellum

71	Left	Thalamus
72	Left	Caudate
73	Left	Putamen
74	Left	Pallidum
75	Left	Hippocampus
76	Left	Amygdala
77	Left	Accumbens nucleus
78	Right	Cerebellar white matter
79	Right	Cerebellum
80	Right	Thalamus
81	Right	Caudate
82	Right	Putamen
83	Right	Pallidum
84	Right	Hippocampus
85	Right	Amygdala
86	Right	Accumbens nucleus
87	-	Brainstem
