# Supplementary material for: Exploring the heterogeneous morphometric data in essential tremor with probabilistic modelling
Source: Neuroimage Clin. 2022 Dec 6;37:103283. doi: 10.1016/j.nicl.2022.103283 (PMC9755240; doi:10.1016/j.nicl.2022.103283)
Supplement: Supplementary data 2 [file mmc2.pdf]

Supplementary Material for *Exploring the heterogeneous morphometric data in essential tremor with probabilistic modelling*

Thomas A.W. Bolton[1,2], Dimitri Van De Ville[3,4], Jean Régis[5], Tatiana Witjas[6], Nadine Girard[7], Marc Levivier[1,8] and Constantin Tuleasca[1,8,9]

This Supplementary Material document is organized as follows: in Section 1, we briefly situate Gamma Knife stereotactic radiosurgical thalamotomy within the most popular existing interventional approaches for severe essential tremor (ET). We then provide methodological details regarding how it was performed for the patients analyzed in the present study.

In Section 2, we recall how a Gaussian Mixture Model can be solved using the expectation-maximization algorithm and specify the parameters that we employed towards this aim in this work.

1. Gamma Knife stereotactic radiosurgical thalamotomy

As many as 50% of patients suffering from ET cannot tolerate commonly prescribed medications or show refractory disabling tremor despite receiving a recommended dose (Elias et al., 2013). In such severe cases, several interventional approaches are available, all of which can target (aside from other candidate areas) the contralateral ventro-intermediate nucleus of the thalamus (Vim) to lower tremor.

The most popular options include deep brain stimulation (DBS), a neuromodulation technique relying on high-frequency electrical stimulation through deeply implanted electrodes. DBS was proven safe and efficient in the treatment of ET, with the specific benefits to interrupt or fine-tune stimulation if required, but risks (e.g., infection) resulting from the required operation and the reliance on implanted hardware (Benabid et al., 1996; Flora et al., 2010). Magnetic resonance-guided focused ultrasound

(MRgFUS) thalamotomy is a more recent lesion-based approach that does not necessitate an invasive operation and provides a risk/benefit balance like more established approaches (Elias et al., 2013; Rohani and Fasano, 2017). Finally, Gamma Knife stereotactic radiosurgical thalamotomy is also a lesion-based approach in which the Vim is located using stereotactic coordinates, avoiding the need for an open operation, and precisely targeted by a beam of gamma radiations; it is a good choice for patients with medical comorbidities or uneasy with the prospect of an operation, but the target site cannot be confirmed intraoperatively and clinical benefits only appear after a few months (Witjas et al., 2016). Many reports have confirmed Gamma Knife thalamotomy to be a safe and effective noninvasive surgical strategy (Kondziolka et al., 2008; Young et al., 2010; Kooshkabadi et al., 2013; Niranjan et al., 2017), and the current literature positions all three as viable options, with similar ranges of clinical improvement and little permanent damages (Dallapiazza et al., 2018).

In the present work, the morphometric properties of severely impaired, drug-refractory ET patients were contrasted to those of matched healthy controls (HCs). While the analyzed imaging data was obtained before any intervention was carried out, a second imaging session was also conducted after each ET patient underwent Gamma Knife stereotactic radiosurgical thalamotomy of the left Vim. In data preprocessing (subsection **Regression of covariates of no interest** of the main content), post-thalamotomy scans were used for the removal of confounding variables (age, gender and total grey matter volume), in order to model within-subject variance on top of cross-subject variability.

We now provide details regarding the Gamma Knife stereotactic radiosurgical thalamotomy process. It was performed using Leksell Gamma Knife (Elekta Instruments, AB, Sweden) between September 2014 and April 2016, at the Centre Hospitalier Universitaire de la Timone (Marseille, France), always by the same neurosurgeon (J.R.). In each case, the Leksell coordinate G frame (Elekta Instruments, AB, Sweden) was applied under local anesthesia on the day of the thalamotomy. After its positioning, stereotactic computed tomography and MRI were both performed on the patient.

Landmarks of interest, such as the anterior and posterior commissures, were identified on an MR scan (T2 CISS/FIESTA sequence, Siemens). Targeting was achieved with

the Guiot diagram (Guiot et al., 1962), placed 2.5 mm above the anterior-posterior commissure line and 11 mm lateral to the third ventricle wall. A single 4-mm isocenter was used with a maximum prescription dose of 130 Gy.

2. Expectation-maximization algorithm to solve a GMM

A dataset exhibiting a multimodal data structure can be represented as a Gaussian mixture model (GMM). We define K as the number of mixed Gaussians. If $\boldsymbol{x}_s$ is a realization from a GMM, we have:

$$\boldsymbol{x}_s \sim \mathcal{G}\left(\{\boldsymbol{\mu}_k, \boldsymbol{\Sigma}_k\}_{k=1,\ldots,K}, \boldsymbol{\pi}\right) \leftrightarrow p\left(\boldsymbol{x}_s | \{\boldsymbol{\mu}_k, \boldsymbol{\Sigma}_k\}_{k=1,\ldots,K}, \boldsymbol{\pi}\right) = \sum_{k=1}^{K} \pi_k \mathcal{N}(\boldsymbol{x}_s | \boldsymbol{\mu}_k, \boldsymbol{\Sigma}_\mathrm{k}).$$

In the above, $\mathcal{G}$ denotes the mixture of Gaussians, each parameterized by a mean vector and a covariance matrix. $\boldsymbol{\pi}$ is a K-element vector that summarizes the respective weighting of the Gaussians and satisfies $\sum_{k=1}^{K} \pi_k = 1$.

Given a set of S independent data points $\mathbf{X}$, the expectation-maximization (EM) algorithm can be used to solve the GMM. First, parameters are initialized: here, for all $k = 1, \ldots K$, we set $\boldsymbol{\Sigma}_k^{(0)}$ to the full dataset's covariance matrix, and $\boldsymbol{\mu}_k^{(0)}$ as a randomly selected data point. We further set $\pi_k^{(0)} = \frac{1}{K}$.

Given a set of defined parameters at iteration i, the *expectation step* involves the computation of each datapoint's *responsibility* $\boldsymbol{\gamma}_s$ as:

$$\gamma_{s,k}^{(i)} = \frac{\pi_k^{(i)} \mathcal{N}_k\left(\boldsymbol{\mu}_k^{(i)}, \boldsymbol{\Sigma}_\mathrm{k}^{(i)}\right)}{\sum_{k=1}^{K} \pi_k^{(i)} \mathcal{N}_k\left(\boldsymbol{\mu}_k^{(i)}, \boldsymbol{\Sigma}_\mathrm{k}^{(i)}\right)} \text{ for } k = 1, \ldots, K.$$

Put simply, the responsibility quantifies to which of the Gaussian modes the data point at hand is the closest, given known underlying parameters. Then, in the *maximization step*, the parameters can be recomputed as:

$$
\begin{cases}
\pi_k^{(i+1)} = \dfrac{\sum_{s=1}^{S} \gamma_{s,k}^{(i)}}{S} \\
\boldsymbol{\mu}_k^{(i+1)} = \dfrac{\sum_{s=1}^{S} \gamma_{s,k}^{(i)} \boldsymbol{x}_s}{\sum_{s=1}^{S} \gamma_{s,k}^{(i)}} \\
\boldsymbol{\Sigma}_k^{(i+1)} = \dfrac{\sum_{s=1}^{S} \gamma_{s,k}^{(i)} \left(\boldsymbol{x}_s - \boldsymbol{\mu}_k^{(i+1)}\right)^{\mathrm{T}} \left(\boldsymbol{x}_s - \boldsymbol{\mu}_k^{(i+1)}\right)}{\sum_{s=1}^{S} \gamma_{s,k}^{(i)}}
\end{cases}
\quad \text{for } k = 1, \dots, K.
$$

The mean vector and the covariance matrix for each Gaussian are simply versions of the multivariate Gaussian estimates reweighted by the responsibilities. At each iteration, the LL of the dataset can also be evaluated as:

$$
\mathcal{LL}_{\mathcal{G}}^{(i)}(\mathbf{X}) = \sum_{s=1}^{S} \log\left(\sum_{k=1}^{K} \pi_k^{(i)} \mathcal{N}\left(\boldsymbol{x}_s | \boldsymbol{\mu}_k^{(i)}, \boldsymbol{\Sigma}_{\mathrm{k}}^{(i)}\right)\right).
$$

The EM process is repeated until convergence of the log-likelihood is achieved, at a tolerance $\tau = 10^{-5}$. To avoid converging on a local optimum, the EM scheme is run 100 separate times with different initializations, and the solution with highest LL is retained.

References

Benabid, Alim Louis, et al. "Chronic electrical stimulation of the ventralis intermedius nucleus of the thalamus as a treatment of movement disorders." *Journal of neurosurgery* 84.2 (1996): 203-214.

Dallapiazza, Robert Francis, et al. "Outcomes from stereotactic surgery for essential tremor." *Journal of Neurology, Neurosurgery & Psychiatry* 90.4 (2019): 474-482.

Elias, W. Jeffrey, et al. "A pilot study of focused ultrasound thalamotomy for essential tremor." *New England Journal of Medicine* 369.7 (2013): 640-648.

Flora, Eliana Della, et al. "Deep brain stimulation for essential tremor: a systematic review." *Movement disorders* 25.11 (2010): 1550-1559.

Guiot, G., et al. "Délimitation précise des structures sous-corticales et identification de noyaux thalamiques chez l'homme par l'électrophysiologie stéréotaxique." *Neurochirurgia* 5.01 (1962): 1-18.

Kondziolka, Douglas, et al. "Gamma Knife thalamotomy for essential tremor." *Journal of neurosurgery* 108.1 (2008): 111-117.

Kooshkabadi, Ali, et al. "Gamma Knife thalamotomy for tremor in the magnetic resonance imaging era." *Journal of neurosurgery*118.4 (2013): 713-718.

Niranjan, Ajay, et al. "Stereotactic radiosurgery for essential tremor: Retrospective analysis of a 19-year experience." *Movement Disorders* 32.5 (2017): 769-777.

Rohani, Mohammad, and Alfonso Fasano. "Focused ultrasound for essential tremor: review of the evidence and discussion of current hurdles." *Tremor and Other Hyperkinetic Movements* 7 (2017).

Witjas, T., et al. "Essential tremor: update of therapeutic strategies (medical treatment and gamma knife thalamotomy)." *Revue Neurologique* 172.8-9 (2016): 408-415.

Young, Ronald F., et al. "Gamma Knife thalamotomy for treatment of essential tremor: long-term results." *Journal of neurosurgery* 112.6 (2010): 1311-1317.
